# Analysis of lifetime death probability for major causes of death among residents in China

**DOI:** 10.1186/s12889-020-09201-7

**Published:** 2020-07-11

**Authors:** Ping Yuan, Jianjun Xiang, Matthew Borg, Tiehui Chen, Xiuquan Lin, Xiane Peng, Kuicheng Zheng

**Affiliations:** 1 Fujian Center for Disease Control and Prevention, No.76 Jintai Road, Gulou District, Fuzhou, 350001 Fujian Province China; 2 Educational Base, School of Public Health, Fujian Medical University, Fuzhou, 350005 Fujian Province China; 3 School of Public Health, The University of Adelaide, Adelaide, SA 5005 Australia

**Keywords:** Lifetime death probability, Chronic diseases, Cause of death, Mortality

## Abstract

**Background:**

Cumulative mortality rate and cumulative mortality risk are two commonly used indicators to measure the impact and severity of diseases. However, they are calculated during a defined life span and assume the subject does not die from other causes. This study aims to use a new indicator, lifetime death probability (LDP), to estimate the lifetime death probabilities for the top five leading causes of death in China and explore the regional differences and trends over time.

**Methods:**

LDPs were calculated using a probability additive formula and abridged life tables.

**Results:**

In 2014, LDPs for heart disease, cerebrovascular disease, malignancy, respiratory disease, and injury and poisoning were 24.4, 23.7, 19.2, 15.5, and 5.3%, respectively. The LDPs for heart disease and malignancy increased by 7.3 and 0.5%, respectively, compared to those from 2004 to 2005. In contrast, the LDPs for cerebrovascular and respiratory disease decreased by 1.0 and 3.9%, respectively, compared to those in 2004–2005. Across the eastern, central and western regions, malignancy had the highest LDP in the eastern region, cerebrovascular and heart diseases in the central region, and respiratory diseases, and injury and poisoning in the western region.

**Conclusions:**

LDP is an effective indicator for comparing health outcomes and can be applied for future disease surveillance. Heart disease and malignancy were the two most common causes of death in China, but with regional differences. There is a need to implement targeted measures to prevent chronic diseases in different regions.

## Background

With the rapid socioeconomic development in the past decades in China and its consequent changes in lifestyle and living environment, chronic diseases have gradually replaced infectious diseases and malnutrition as the leading cause of mortality [1]. According to the latest national statistics, the top five leading underlying causes of death in 2014 were malignancy, cerebrovascular disease, heart disease, respiratory disease, and injury and poisoning [2]. They resulted in substantial years of life lost (YLLs) and disability-adjusted life years (DALYs) [3], affecting population health and burdening the overloaded healthcare system. The number of DALYs from malignancy was as high as 1893 per 100,000 in the 15–49 age group [4].

Measuring the mortality from major causes of death can help explain changes in population health, evaluate health strategies and performance, and guide policy-making. Cumulative mortality rate and cumulative mortality risk are two commonly used indicators to measure the impact and severity of diseases [5, 6]. Cumulative mortality risk is the probability that an individual will die from a particular disease during a specified time period, assuming that he/she does not suffer from other causes [7]. Cumulative mortality rate is the sum of the age-specific mortality rates from each age band within a pre-defined time period. It is based on multiplicative model of probability theory [8]*.* Cumulative mortality rate is also an indicator of death probability from a cause before a certain age, commonly expressed as a percentage and used as an approximation of the cumulative mortality risk [9, 10]. It is easy to convert a cumulative rate to a cumulative mortality risk [11]. Whilst not influenced by population composition, these two indicators can only be calculated in subjects without a competing risk of death and only in certain age groups [12]. For example, Hao [13] reported that the cumulative mortality risk for malignancies was 13% before the age of 74 years. However, if one’s life expectancy was longer than 74 years, the probability of death could not be estimated.

A new method, lifetime death probability (LDP), based on cumulative risk, was introduced to estimate the likelihood of death throughout individuals’ lifetimes using data from our previous study [8, 12, 14, 15]. LDP considers the competing risk of death and is unaffected by demographic composition. This study aimed to (1) calculate the LDP for the top 5 leading causes of death in mainland China; and (2) explore the regional differences and trends over time. This knowledge could provide evidence to support the development of chronic disease prevention strategies, performance evaluation, and the allocation of healthcare resources for targeted interventions.

## Methods

### Data sources

The age-specific mortality rate from 1st January 2004 to 31st December 2005 was sourced from the findings of the Third National Death Cause Survey [16]. This survey covered 213 counties across China and was hosted by the Ministry of Health of the People’s Republic of China. The age-specific mortality rates for 2014 were extracted from the Chinese National Death Cause Monitoring Dataset [2]. This dataset was compiled by the National Health and Family Planning Commission of the People’s Republic of China using 605 national disease surveillance system monitoring stations. China has used these two systems, the survey findings and dataset, for decades to provide nationally representative data on health status to guide health-care decision-making and performance evaluation. However, these systems overlapped to a considerable extent, entailing a duplication of effort. In 2013, the Chinese Government combined these two systems into an integrated national mortality surveillance system. This system provides a provincially representative picture of total and cause-specific mortality with which to accelerate the development of a comprehensive, nation-wide, system for vital registration and mortality surveillance. This new system increased the Chinese surveillance population from 6 to 24% [17].

Malignancy (C00-C97), cerebrovascular disease (I60-I69), heart disease (I05-I09, I10–13, I20–25), respiratory disease (J30-J98) and injury and poisoning (V01-Y89) were classified using the International Classification of Diseases, 10th revision (ICD-10) (WHO, 1992).

### Statistical analysis

Based on the probability additive formula [12, 14], an abridge life table [9] was used to calculate the LDPs for the five leading causes of disease. The series of formulas is as follows:

1 $$ {q}_x=\sum \limits_i{q}_x^i $$

2 $$ {D}_x=\sum \limits_i{D}_x^i $$

3 $$ {r}_x^i=\frac{D_x^i}{D_x}=\frac{m_x^i}{m_x} $$

4 $$ {q}_x^i={r}_x^i{q}_x $$

5 $$ {d}_x^i={r}_x^i{d}_x $$

6 $$ {P}_x^i=\frac{\sum \limits_x^w{d}_x^i}{l_x} $$

7 $$ {P}_0^i=\frac{\sum \limits_{x=0}^w{d}_x^i}{l_0} $$

*q* is the death probability. Subscript *x* and superscript *i* are indicator variables representing the age group and cause of death, respectively. D is the number of deaths. *m* is the mortality rate. *r* is the proportional mortality. *d* and *l* designate the death toll and survival number, respectively, in the life table. *w* is an indicator variable representing the oldest age group. ${P}_0^i$ of age group 0 is an individual’s LDP [8].

China is a vast country with varying socioeconomic development status in different regions. Furthermore, trends in mortality and disease burden differ significantly at a provincial level. To facilitate the identification of health priorities at a regional level, we calculated the region-specific lifetime death probabilities for the first five major causes of death [3]. According to the National Bureau of Statistics, China can be divided geographically into three regions: the eastern, central, and western regions [14]. The eastern region includes the following municipalities and provinces: Beijing, Tianjin, Hebei, Liaoning, Shanghai, Jiangsu, Zhejiang, Fujian, Shandong, Guangdong, and Hainan; the central region includes Heilongjiang, Jilin, Shanxi, Anhui, Jiangxi, Henan, Hubei, and Hunan; and the western region includes Inner Mongolia, Guangxi, Chongqing, Sichuan, Guizhou, Yunnan, Tibet, Shaanxi, Gansu, Qinghai, Ningxia, and Xinjiang.

## Results

From 2004 to 2005, there were 868,484 deaths across the monitoring areas of China. Males and females accounted for 57.9% (502,434) and 42.1% (366,050) of deaths, respectively. The death toll was 331,521 (38.2%) in eastern China, 299,406 (34.5%) in central China and 237,557 (27.4%) in western China, respectively (data not shown). In 2014, the total mortality count in the monitoring areas increased to 1,643,377, with males and females accounting for 58.7% (965,261) and 41.3% (678,116) of deaths, respectively. Specifically, the death toll was 656,709 (40.0%) in eastern China, 565,285 (34.4%) in central China and 421,383 (25.6%) in western China, respectively (data not shown).

Table 1 shows example calculations for cerebrovascular disease based on the life table method. The LDP of cerebrovascular disease in 2014, ${P}_0^i$, was 23.7% (0.237). Similarly, the LDPs in 2014 for heart disease, respiratory disease, malignancy and injury and poisoning were 24.4, 19.2, 15.5, and 5.3%, respectively (Table 2). And the LDPs in 2004–2005 for cerebrovascular disease, respiratory disease, malignancy, heart disease and injury and poisoning were 24.7, 19.4, 18.7, 17.1 and 6.5%, respectively (Table 3).

**Table 1:** Calculation on LDP of cerebrovascular disease in 2014, China

*x* (1)	*m*_*x*_ (2)	${m}_x^i$ (3)	*q*_*x*_ (4)	*l*_*x*_ (5)	*d*_*x*_ (6)	${r}_x^i$ (7) = (3)/(2)	${d}_x^i$ (8) = (6)*(7)	$\sum \limits_x^w{d}_x^i$ (9)	${P}_x^i$ (10) = (9)/(5)
0~	460.6	0.000	0.005	100,000	461	0.000	0	23,743	0.237
1~	50.0	0.000	0.002	99,539	199	0.002	0	23,742	0.239
5~	21.6	0.000	0.001	99,341	107	0.009	1	23,742	0.239
10~	26.1	0.000	0.001	99,233	130	0.012	2	23,741	0.239
15~	35.9	0.000	0.002	99,104	178	0.032	6	23,740	0.240
20~	37.7	0.000	0.002	98,926	186	0.040	7	23,734	0.240
25~	62.7	0.000	0.003	98,740	309	0.043	13	23,727	0.240
30~	85.2	0.000	0.004	98,430	419	0.073	30	23,713	0.241
35~	104.2	0.000	0.005	98,012	509	0.094	48	23,683	0.242
40~	174.4	0.000	0.009	97,503	847	0.122	103	23,635	0.242
45~	230.0	0.000	0.011	96,656	1105	0.144	159	23,531	0.243
50~	437.1	0.001	0.022	95,551	2066	0.171	353	23,372	0.245
55~	557.5	0.001	0.027	93,485	2570	0.180	462	23,018	0.246
60~	1014.7	0.002	0.049	90,915	4498	0.202	907	22,557	0.248
65~	1755.2	0.004	0.084	86,417	7265	0.232	1682	21,650	0.251
70~	2820.0	0.007	0.132	79,152	10,425	0.252	2625	19,968	0.252
75~	4367.1	0.012	0.197	68,727	13,530	0.283	3833	17,343	0.252
80~	8051.6	0.021	0.335	55,197	18,498	0.266	4917	13,509	0.245
85+	16,640.0	0.039	1.000	36,699	36,699	0.234	8592	8592	0.234

Note: see the method section for the explanation of each index in row headings

**Table 2:** Mortality and LDP for the five leading causes of death by region, 2014, China

Top 5 leading causes of death	Overall		Eastern region		Central region		Western region	
	Mortality rate^a^	LDP%	Mortality rare^a^	LDP%	Mortality rare^a^	LDP%	Mortality rare^a^	LDP%
Malignancy	155.4	19.2	175.4	21.2	144.7	18.3	139.2	16.7
Cerebrovascular disease	143.6	23.7	142.5	23.0	149.0	24.9	137.6	22.5
Heart disease	141.3	24.4	144.9	23.9	151.4	27.3	121.6	20.3
Respiratory disease	78.2	15.5	70.9	13.7	64.3	13.2	108.7	21.4
Injury and poisoning	49.7	5.3	45.7	5.1	48.1	5.1	58.1	5.7

^a^Mortality rate per 100,000 population

**Table 3:** Mortality and LDP for the five leading causes of death by region, 2004–2005, China

Top 5 leading causes of death	Overall		Eastern region		Central region		Western region	
	Mortality rate^a^	LDP%	Mortality rate^a^	LDP%	Mortality rare^a^	LDP%	Mortality rare^a^	LDP%
Malignancy	135.9	18.7	154.7	20.2	135.9	19.3	111.4	15.8
Cerebrovascular disease	136.6	24.7	146.6	25.2	146.8	26.4	111.2	21.8
Heart disease	90.2	17.1	96.8	17.3	99.7	19.0	70.0	14.0
Respiratory disease	96.3	19.4	91.2	17.9	84.1	17.2	117.9	24.7
Injury and poisoning	61.5	6.5	58.5	6.3	60.1	6.6	67.2	6.7

^a^Mortality rate is per 100,000 population

The order of the first five causes of death had changed by using LDPs compared to mortality rates. As shown in Table 2, malignancy had the highest mortality rate in 2014, followed by cerebrovascular disease, heart disease, respiratory disease, and injury and poisoning. However, according to the LDPs, heart disease was the first leading cause of death, followed by cerebrovascular disease, malignancy, respiratory disease and injury and poisoning. The situation in 2004–2005 was similar to that in 2014. As shown in Table 3, cerebrovascular disease had the highest mortality rate from 2004 to 2005 in China, followed by malignancy, respiratory disease, heart disease, and injury and poisoning. However, according to the LDPs, cerebrovascular disease was the first leading cause of death, followed by respiratory system disease, malignancy, heart disease and injury and poisoning. Specifically, from 2004 to 2005 to 2014, the LDPs of heart disease and malignancy had increased by 7.3 and 0.5%, respectively. In contrast, LDPs due to respiratory disease, injury and poisoning, and cerebrovascular disease decreased by 3.9, 1.2, and 1.0%, respectively.

We also observed that there were regional differences in the order of the most common cause of death expressed by different indicators (mortality rate and LDP). In the eastern region, the most common cause of death is malignancy in two different periods (2004–2005 and 2014) indicated by mortality rate, but in the case of LDP, the most common cause of death is cerebrovascular disease in 2004–2005 and heart disease in 2014. In the western region, the most common cause of death is malignancy in 2014 with mortality rate, but the most common cause of death with LDP is cerebrovascular disease in 2014. Different from the eastern and western regions, the rank order of causes of death by mortality rates is the same as LDP in the central region. Across the eastern, central and western regions, malignancy had the highest LDP in the eastern region, cerebrovascular and heart diseases in the central region, and respiratory diseases, and injury and poisoning in the western region.

## Discussion

To our best knowledge, this is the first epidemiological study using LDP to evaluate the severity and risk for the five leading underlying causes of death in China. Comparing regional differences and changes over time can provide insights into current priorities for disease prevention in different regions.

### LDPs for the top five leading causes of death: implications for priority identification

Although malignancy was the most common cause of death, the probability of death due to malignancy in a person’s lifetime was lower than that due to cerebrovascular and heart disease, the second and third most common causes of death in 2014, respectively. This phenomenon could be due to the differences in mortality rate between the diseases in certain age groups. The mortality rate of malignancy (54/100,000 population) was more than two times higher than that of cardiovascular (20/100,000 population) and cerebrovascular disease (21/100,000 population) at the age of 40–45 [2]. However, at the age of 80–85, the mortality rate of cancer (1266/100,000 population) was much lower than that of cardiovascular (2066/100,000 population) and cerebrovascular disease (2140/100,000 population) [2]. In the presence of competing risks, the events of interest are excluded by different events that had occurred previously [18]. Suppose now that the event of interest is the onset of “given disease”. Individuals may die without developing the “given disease”. We may then be interested in the risk or probability of getting the disease in a given follow-up period [5]. Calculation of the mortality rate is based on observed population without the disease as “independent censoring”, that is, a purely hypothetical population where individuals could not die without the disease. LDP is a much more satisfactory indicator, where one acknowledges that individuals may die without the disease and where inference for disease risks and rates are made “in the presence of the competing risk of dying”. In terms of age-specific mortality, those who died of cancer were younger, but those who died of cardiovascular and cerebrovascular diseases were older. On the surface, the mortality rate of malignancy is higher than that of cardiovascular and cerebrovascular diseases when not considering competing risks. The competing risk of death may be partly responsible for the observed difference between mortality rates and the LDPs. Furthermore, another important contributor is likely to be the current age structure of the Chinese population, which will influence the average mortality rate, but not the LDP. Therefore, we should consider the impact of competing risks and the age structure when assessing the severity of the disease. In this study, when formulating disease prevention policies, we should not only focus on the prevention of tumors, but also on the prevention of cardiovascular and cerebrovascular diseases.

### Change of trends in LDP

Compared to 2004–2005, we found the LDPs from heart disease and malignancy increased in 2014, while the LDPs for respiratory disease and injury and poisoning in a person’s lifetime decreased. The Chinese government at all levels has actively promoted prevention measures to effectively control major risk factors in recent years. Respiratory disease, cerebrovascular disease and injuries and poisoning have declined, but heart disease and malignancy are still increasing [19]. These changes may require an integrated government response to improve primary health care and address key risks for heart disease and malignancy. To curb the increasing trend in chronic diseases (e.g. heart disease and malignancy), the Chinese central government has launched the initiative “Medium-to-Long Term Plan of China for the Prevention and Treatment of Chronic Diseases (2017–2025)” in 2017 [20]. Recommended prevention strategies include promoting health education, enforcing early diagnosis, reinforcing standardized treatment, strengthening the cooperation between health organizations, reforming healthcare policies, and improving chronic disease surveillance.

### Regional differences

In this study, we found that the LDPs for the top five leading causes of death varied by regions. Across the eastern, central and western regions, malignancy had the highest LDP in the eastern region, cerebrovascular and heart diseases in the central region, and respiratory diseases, and injury and poisoning in the western region. A wide range of factors may contribute to the differences between regions including different lifestyles, eating habits, geographical locations, living environments, socioeconomic status, availability of medical resources, and access to medical screening and treatment. The eastern region is well developed, with a faster working pace and greater work pressure than other two regions, and possibly increased medical access to screening and treatment for cancer [21–23]. It is of great importance in etiology and public health to explore the generality and characteristics of interregional disease incidence and mortality.

Factors contributing to obesity include rapid economic development and urbanization process, and unhealthy diets and lifestyles. Obesity is associated with heart disease and cerebrovascular disease [24, 25]. Proposed mechanisms linking obesity to cardiovascular and cerebrovascular disease include insulin resistance and chronic subclinical inflammation [26]. In recent years, the prevalence for obesity in the central region has been on the rise and increasing at a faster rate than the rate in eastern and western regions [27]. This could explain why the number of hypertension cases and deaths from hypertensive diseases are significantly higher in the central region than in the eastern and western regions [28]. In contrast, the LDP for respiratory disease was the highest in the western regions compared to the other regions. Relatively speaking, the western region has a lagging economy small population, and poor and uneven distribution of medical resources. The common use of indoor coal may be a causative factor for the high mortality rates from respiratory disease in this region [29–31]. In addition, a higher smoking rate may also contribute to the higher LDP in the western region compared to other two regions [32].

### Strengths and limitations

In this study, we used LDP as an indicator to estimate the death probability for the top five leading causes of death. Compared to cumulative mortality rate and cumulative mortality risk, LDP has its advantages. Firstly, LDP can calculate the probability of death in one’s lifetime by using a probability addition model. However, cumulative mortality rate or cumulative mortality risk only be calculated for a specific age range (usually 0–74 years old) by using a probability multiplicative model. Secondly, LDP, based on the abridged life tables, is not affected by population composition [12]. Finally, LDP is a satisfactory indicator, where inference for disease risks and rates are made “in the presence of the competing risk of dying”. Although cumulative mortality rate or cumulative mortality risk can indicate the severity of a certain death cause, they do not take into account the existence of multiple causes of death. Therefore, LDP may be a more appropriate statistical indicator for situations where multiple causes of death and competing risks could be considered.

This study has potential limitation, and caution should be exercised in interpreting the results. The number of mortality monitoring points in China increased from 213 in 2004–2005 to 605 in 2014, which may affect the consistency in estimating the age-specific mortality rates. Nevertheless, in 2013 the National Health and Family Planning Commission combined the vital registration system and the disease surveillance points system to create an integrated national mortality surveillance system [2]. As the two mortality surveillance systems were similar in disease coding and classification, sampling method, and regional divisions, the age-specific mortality rates between 2004 and 2005 and 2014 should be comparable.

## Conclusions

LDP is an effective indicator for comparing health outcomes and can be applied in future disease surveillance. Heart disease and cancer are the two most likely causes of death in China and both have regional differences. There is a need to take targeted preventive measures to prevent chronic diseases in different regions.

## Supplementary information

**Additional file 1.** Age-specific mortality rate in different areas in 2004 and 2014, China.

## Data Availability

The data analyzed in the current study are available from publicly available books. The data has been entered into an Excel file which is included as a supplementary file.

## Abbreviations

LDP: Lifetime death probability
YLL: Years of life lost
DALY: Disability-adjusted life years
